# Cardiovascular Disease and Fracture Risk in People with Type 2 Diabetes: A Nationwide Matched Case–Control Study

**DOI:** 10.1007/s00223-026-01556-0

**Published:** 2026-05-29

**Authors:** Aksayan Arunanthy Mahalingasivam, Annika Vestergaard Kvist, Joop P. van den Bergh, Cassandra Smith, Joshua R. Lewis, Peter Vestergaard, Nicklas Højgaard-Hessellund Rasmussen

**Affiliations:** 1 Steno Diabetes Centre North Denmark, Aalborg University Hospital, Aalborg, Denmark; 2 Department of Clinical Medicine, Aalborg University, Aalborg, Denmark; 3 VieCuri Medical Center, Maastricht University Medical Center, Maastricht, The Netherlands; 4 Nutrition & Health Innovation Research Institute, School of Medical and Health Sciences, Edith Cowan University, Perth, WA Australia; 5 Medical School, The University of Western Australia, Perth, WA Australia

**Keywords:** Diabetes mellitus type 2, Cardiovascular disease, Fracture, Co-morbidities, Medication

## Abstract

To examine, among people with type 2 diabetes (T2D), whether cardiovascular disease (CVD) phenotypes and cardiovascular medications are associated with any fracture as the primary outcome and major osteoporotic fracture (MOF) as a secondary outcome. Nationwide registry-based matched case–control study in Denmark (2013–2021) among people with T2D. Cases were individuals with T2D who sustained an incident fracture (n = 12,390), and controls were fracture-free individuals with T2D matched 1:3 by age, sex, T2D onset date, and weighted Charlson Comorbidity Index (n = 37,170). The exposures were overall CVD, prespecified clinical CVD phenotypes (acute myocardial infarction (AMI), stroke, heart failure, atrial fibrillation/flutter (AFib/AFL), and atherosclerosis), subclinical CVD phenotypes (hypertension, hypercholesterolemia, and chronic kidney disease (CKD)), individual CVD subtypes, and cardiovascular medications. Any fracture was the primary outcome, and major osteoporotic fracture (MOF) was examined as a secondary outcome. Conditional logistic regression estimated overall and sex-stratified odds ratios (ORs) with 95% confidence intervals (CIs), and sex interactions were assessed. any fracture, overall CVD (OR 1.20; 95% CI 1.15–1.26), clinical (OR 1.15; 1.09–1.21), and subclinical CVD (OR 1.22; 1.17–1.28) were associated with higher odds. AFib/AFL, atherosclerosis, CKD, heart failure, hypercholesterolemia, hypertension, and stroke showed positive associations, whereas AMI was inversely associated. Higher fracture odds were observed with factor Xa inhibitors, nitrates, and antiplatelet agents. Sex-stratified analyses showed broadly similar associations for CVD phenotypes in men and women, with no consistent evidence of sex-specific differences. Associations were weaker and non-significant for major osteoporotic fractures. In T2D, CVD and selected cardiovascular medications are associated with higher odds of fracture, highlighting the importance of incorporating cardiovascular status into fracture risk assessment.

**Supplementary Information:**

The online version contains supplementary material available at 10.1007/s00223-026-01556-0.

## Introduction

Type 2 diabetes mellitus (T2D) is characterized by insulin resistance and dysregulated glucose homeostasis [1]. Chronic hyperglycaemia drives endothelial dysfunction, inflammation, and oxidative stress, accelerating both micro- and macrovascular complications [2]. Among these, cardiovascular disease (CVD) is the most prevalent and remains the leading cause of morbidity and mortality in people with T2D. It commonly coexists with hypertension, dyslipidaemia, and obesity, further amplifying the cardiovascular burden [3, 4].

Sex differences in CVD are well established(5). Men typically develop ischemic heart disease, atherosclerosis, and heart failure at a younger age, whereas women more often present with hypertension, arrhythmia, or stroke later in life(6–8). These differences reflect both biological and hormonal influences as well as disparities in risk factor profiles and clinical presentation [9, 10].

Beyond their vascular manifestations, CVD and associated comorbidities in diabetes are linked to multisystem impairments [11]—including neuropathy, balance dysfunction, and medication effects [12]—that compromise physical function and postural stability, thereby increasing fall risk [13, 14]. Among people with T2D, coexisting CVD further increases this susceptibility, but the extent to which discrete CVD subtypes contribute to fracture risk remains unclear [15]. Understanding these relationships is clinically relevant, as different cardiovascular phenotypes may underlie distinct fall mechanisms (e.g., arrhythmia or post-stroke sequelae) and skeletal alterations (e.g., chronic kidney disease—mineral and bone disorder). Cardiovascular disease can be conceptualized as a continuum, ranging from early vascular dysfunction and end-organ damage to overt clinical events [16]. Conditions such as hypertension, hypercholesterolemia, and chronic kidney disease (CKD) reflect established vascular pathology [16], while CKD is closely linked to endothelial dysfunction and accelerated atherosclerosis [17], even in the absence of acute cardiovascular events [18].

People with T2D also display intrinsic skeletal fragility [19]. Proximal mechanisms include impaired postural control and neuropathy-related deficits in sensory-motor integration, whereas distal skeletal mechanisms include diabetes-related deterioration in body composition [20] and bone quality and microarchitecture, rendering the skeleton more fragile than in people without T2D [21, 22]. Cardiovascular disease, as a common comorbidity in T2D, may further exacerbate these mechanisms through shared vascular–skeletal pathways, motivating examination of its association with fracture risk [23]. Paradoxically, although people with T2D often have normal or higher bone mineral density, partly related to higher body mass index, this does not translate into lower fracture risk [24, 25]. Instead, diabetes-related skeletal fragility may involve impaired bone quality, including higher cortical porosity, together with poorer physical function and increased fall susceptibility(24, 26). Collectively, the abovementioned deficits lead to people living with T2D having higher fracture risk than those without with studies finding a pooled relative risk of 1.38 in people living with T2D [25].

Together, these mechanisms suggest that cardiovascular disease may be an important marker of fracture risk in people with T2D. The present study therefore aimed to examine, among people with T2D, whether CVD phenotypes were associated with any fracture as the primary outcome and major osteoporotic fracture (MOF) as a secondary outcome, and to identify which CVD subtypes were most strongly associated with fracture risk. In additional analyses, we examined whether use of commonly prescribed cardiovascular medications was associated with these fracture outcomes. Given known sex differences in both cardiovascular disease patterns and fracture risk, all analyses were stratified by sex.

## Method

### Data source

Data were obtained from Statistics Denmark (project number 703382) and included the Danish National Patient Registry, which has collected nationwide hospital diagnoses coded in ICD-10 (and historically ICD-8) since 1977. Medication data were retrieved from the Register of Medicinal Products Statistics, containing all prescription drugs dispensed at Danish pharmacies and coded using the Anatomical Therapeutic Chemical (ATC) classification since 1996. These registers were linked using the unique civil personal registration (CPR) number assigned to all Danish residents.

### Ethics

This study was based on anonymized data from nationwide Danish health registers. No direct participant involvement occurred, and ethical approval and informed consent were therefore not required under Danish law.

### Study Design and Population

We conducted a nationwide registry-based case–control study including people above 18 years of age with T2D between January 1, 2013, and December 31, 2021. The study period refers to the period during which incident fractures and corresponding index dates were identified, whereas T2D could have been diagnosed before or during this period.

People with T2D were identified by a first recorded diagnosis code of T2D (ICD-10 code E11, E13–E14 and/or ICD-8 code 250) or by redeemed antidiabetic prescription (ATC codes A10AE54, A10AE56 or A10B; excluding Saxenda). ICD-8 codes were included to capture historical T2D diagnoses recorded before the transition to ICD-10 coding in Denmark, as such diagnoses are not necessarily recoded or updated to ICD-10 in the register history. We excluded people with type 1 diabetes mellitus (ICD-10 code E10 and/or ICD-8 code 249), malnutrition-related diabetes mellitus (ICD-10 code E12), gestational diabetes mellitus (ICD-10 code O24.4) and polycystic ovary syndrome (PCOS) (ICD-10 E28.2; ICD-8 25,690) treated with metformin alone (ATC A10BA02) or in combination with PCOS related medications (ATC G03GB02 or G03HB). People with CVD diagnoses recorded prior to their first documented T2D diagnosis were excluded to ensure a consistent temporal sequence in which T2D preceded CVD exposure. This approach was chosen to minimise ambiguity in exposure classification and to focus on CVD occurring within the course of established T2D.

### Exposure

#### CVD Exposure

CVD exposures were identified using ICD-10 codes recorded after the diagnosis of T2D and before the index date, which was defined as the incident fracture date for cases and the corresponding matched index date for controls (Supplementary Table S2). A composite CVD endpoint was defined and further categorized into clinical and subclinical CVD. Clinical CVD included acute myocardial infarction (AMI), stroke, heart failure, atrial fibrillation/flutter (AFib/AFL), and atherosclerosis, whereas subclinical CVD was operationally defined as hypercholesterolemia, hypertension, and CKD [27]. Each CVD subtype was analysed individually. This grouping was intended to capture cardiovascular risk-related and cardiometabolic–renal conditions that may reflect vascular dysfunction or end-organ involvement preceding overt cardiovascular events; however, these conditions should not be interpreted as manifest clinical CVD.

#### Medication Exposure

Exposure to cardiovascular, diabetes, and osteoporosis medications was identified using ATC codes (Supplementary Table S2). Medication exposure was defined as ≥ 2 redeemed prescriptions within 365 days before the index date. This threshold was chosen to increase the likelihood of capturing ongoing treatment rather than single dispensing and to reduce misclassification related to the initiation phase, during which treatment is frequently modified due to insufficient glycaemic response or adverse effects. While redeemed prescriptions improve the probability of actual medication use, adherence, cumulative duration, and dose intensity could not be directly assessed.

### Outcome

Fractures were identified using ICD-10 fracture diagnoses codes (Supplementary Table S2) excluding fingers, toes and facial fractures, however including osteoporotic and trauma led fractures. To ensure incident events, fractures were validated by confirming the absence of any fracture code affecting the same anatomical region during the preceding 12 months. Any fracture recorded prior to the T2D diagnosis date was documented as a previous fracture for both cases and controls. The index date for cases was defined as the date of the first eligible fracture after their diagnosis of T2D during the study period. For controls without a fracture incident, the index date was set to the corresponding index date of their matched case. Any fracture was defined as the primary outcome. An additional composite outcome, termed major osteoporotic fracture (MOF), was defined as the secondary outcome and included clinically registered vertebral, humerus, forearm, and hip fractures identified using ICD-10 diagnosis codes.

### Covariates

Covariates were identified using ICD-10 codes and ATC codes recorded before the index date (Supplementary Table S2). Comorbidities and diabetes-related complications were defined using ICD-10 codes, and previous fractures were defined as any ICD-10 or ICD-8 registered fracture events before the index date. Overall comorbidity burden was quantified using the weighted Charlson Comorbidity Index (CCI). Age at index was calculated from date of birth.

### Statistical Analysis

Descriptive statistics were calculated for all variables in cases and controls, stratified by sex. Continuous variables were presented as mean ± standard deviation (SD) when normally distributed or as median with interquartile range (IQR) when non-normally distributed. Data distribution was assessed using the Anderson–Darling test. Between-group comparisons used the two-sample t-test for normally distributed continuous variables and the Wilcoxon rank-sum test for non-normally distributed data. Categorical variables were summarized as numbers and percentages [n (%)] and compared using the chi-squared test.

Conditional logistic regression was used to estimate the association between CVD exposures recorded before the index date and subsequent fracture outcomes, including any fracture as the primary outcome and MOF as a secondary outcome. Overall analyses were conducted to estimate the population-level association between CVD phenotypes, cardiovascular medication use, and fracture odds. In addition, all analyses were stratified by sex, as prespecified, to examine whether associations differed between men and women. In secondary analyses, we examined associations between commonly prescribed cardiovascular medications and fracture outcomes. Formal interaction tests were used to assess evidence of sex-specific differences. Results are reported as odds ratios (ORs) with 95% confidence intervals (CIs), using a two-sided significance level of α < 0.05. Given the number of related exposures examined, we did not apply a formal correction for multiple comparisons; results should therefore be interpreted with appropriate caution, with emphasis on consistency and clinical plausibility. Interaction terms were tested to assess whether associations differed by sex, with corresponding z- and p-values. All statistical analyses were conducted in RStudio (version 2025.09.0 + 387) and Microsoft Excel (version 16.101.2).

## Results

### Study Population

**Fig. 1:**
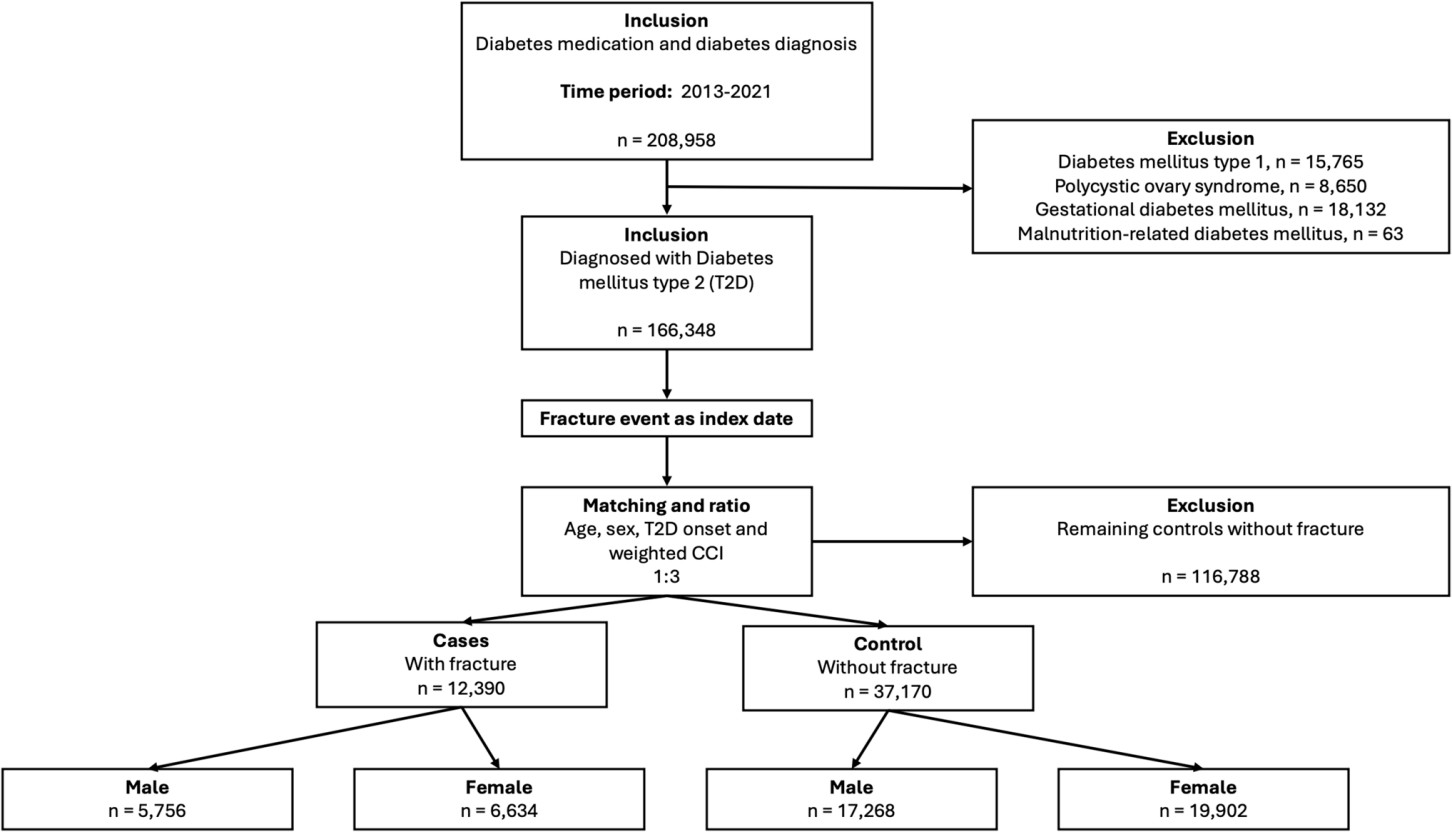
Flowchart of study population identification and selection. People with diabetes were identified between 2013 and 2021. After excluding type 1 diabetes, polycystic ovary syndrome, gestational diabetes, and malnutrition-related diabetes, people with type 2 diabetes (T2D) were matched to controls by age, sex, T2D onset and weighted Charlson Comorbidity Index (CCI) in a 1:3 ratio. Stratification was performed by sex and by exposure status—fracture event

From a cohort of 208,958 people with diabetes, after exclusion of 42,610 with diabetes mellitus type 1, polycystic ovary syndrome, gestational diabetes and malnutrition-related diabetes mellitus, we identified 166,348 people with T2D between 2013–2021. From this pool there were identified 12,390 cases with fractures that were included and matched 1:3 to 37,170 T2D controls without fractures by age, sex, T2D onset date and weighted CCI (Fig. 1), resulting in a remaining 116,788 controls. Matching on weighted CCI was intended to balance overall comorbidity burden between cases and controls. As CCI includes cardiovascular components, this approach may have reduced differences in baseline cardiovascular morbidity between groups; however, CVD exposures were analysed separately at the diagnostic level. Stratification of the study population was performed by sex and by exposure status—fracture event.

### Person characteristics

Women were older than men in both cases and controls. Diabetes duration was similar across sex and groups. Among fracture cases, (50%) sustained a major osteoporotic fracture, and any prior fracture was more common among cases than controls (45% vs 30%). Prevalence of nephropathy and retinopathy was slightly higher in men, whereas neuropathy was comparable between sexes.

CVD prevalence was higher among cases than controls, including composite CVD exposure (75% vs 68%), clinical CVD (28% vs 25%), and subclinical CVD (47% vs 43%). Among individual CVD diagnoses, stroke (14% vs 12%), hypertension (42% vs 38%), CKD (4.2% vs 3.1%), AFib/AFL (5.0% vs 4.2%), heart failure (3.5% vs 3.0%), and hypercholesterolemia (18% vs 17%) were more frequent among cases than controls, whereas AMI was slightly less frequent among cases (8.3% vs 8.9%). Within both cases and controls, AMI was more frequent in men than in women, whereas hypertension was more frequent in women than in men. Heart failure was more frequent in men than in women.

Medication use patterns were largely similar between cases and controls. Statin use was high and comparable (33% vs 32%), while antiplatelet agent use was slightly higher among cases (18% vs 16%). Use of glucagon-like peptide-1 receptor agonists (GLP1-RA) and sodium–glucose cotransporter-2 inhibitors (SGLT2-i) was very low in all groups. Full descriptive characteristics are presented in Table 1. Baseline characteristics and cardiovascular exposure distributions among people with and without MOF are presented in Supplementary Table S1.

**Table 1:** Population characteristics

Variable	People with T2D and fractures (Cases)			People with T2D without fractures (Controls)		
	Men (n = 5,756)	Women (n = 6,634)	Overall (n = 12,390)	Men (n = 17,268)	Women (n = 19,902)	Overall (n = 37,170)
Age, median [IQR]	65.90 [55.03; 75.68]	72.07 [61.22; 80.65]	69.41 [58.05; 78.61]	66.10 [55.31; 75.68]	71.02 [60.77; 79.22]	69.01 [58.12; 77.79]
CCI-weighted, median [IQR]	1.00 [0.00;2.00]	1.00 [0.00;2.00]	1.00 [0.00;2.00]	1.00 [0.00;2.00]	1.00 [0.00;2.00]	1.00 [0.00,2.00]
Any prior fracture, n (%)	2,544 (44%)	3,044 (46%)	5,588 (45%)	5,056 (29%)	6,081 (31%)	11,137 (30%)
MOF, n (%)	2,469 (43%)	3,761 (57%)	6,230 (50%)	283 (1.6%)	748 (3.8%)	1,031 (2.8%)
*Diabetes status*						
Diabetes duration, years, median [IQR]	2.17 [0.89;4.04]	2.26 [0.93;4.05]	2.23 [0.91;4.04]	2.25 [0.94;4.06]	2.38 [1.00;4.18]	2.32 [0.97,4.13]
Nephropathy, n (%)	189 (3.3%)	139 (2.1%)	328 (2.6%)	411 (2.4%)	343 (1.7%)	754 (2.0%)
Neuropathy, n (%)	805 (14%)	930 (14%)	1,735 (14%)	1,706 (9.9%)	2,468 (12%)	4,174 (11%)
Retinopathy, n (%)	209 (3.6%)	224 (3.4%)	433 (3.5%)	523 (3.0%)	587 (2.9%)	1,110 (3.0%)
*Cardiovascular status*						
Composite CVD exposure, n (%)	4,319 (75%)	5,014 (76%)	9,333 (75%)	11,928 (69%)	13,491 (68%)	25,419 (68%)
Clinical CVD, n (%)	1,763 (31%)	1,685 (25%)	3,448 (28%)	4,873 (28%)	4,576 (23%)	9,449 (25%)
Subclinical CVD, n (%)	2,556 (44%)	3,329 (50%)	5,885 (47%)	7,055 (41%)	8,915 (45%)	15,970 (43%)
Atherosclerosis, n (%)	299 (5.2%)	292 (4.4%)	591 (4.8%)	756 (4.4%)	815 (4.1%)	1,571 (4.2%)
AMI, n (%)	621 (11%)	411 (6.2%)	1,032 (8.3%)	2,076 (12%)	1,250 (6.3%)	3,326 (8.9%)
Stroke, n (%)	852 (15%)	899 (14%)	1,751 (14%)	2,089 (12%)	2,334 (12%)	4,423 (12%)
Hypertension, n (%)	2,213 (38%)	3,004 (45%)	5,217 (42%)	6,012 (35%)	8,118 (41%)	14,130 (38%)
CKD, n (%)	274 (4.8%)	247 (3.7%)	521 (4.2%)	568 (3.3%)	583 (2.9%)	1,151 (3.1%)
AFib/AFL, n (%)	293 (5.1%)	324 (4.9%)	617 (5.0%)	763 (4.4%)	795 (4.0%)	1,558 (4.2%)
Heart failure, n (%)	258 (4.5%)	178 (2.7%)	436 (3.5%)	638 (3.7%)	480 (2.4%)	1,118 (3.0%)
Hypercholesterolemia, n (%)	1,122 (19%)	1,157 (17%)	2,279 (18%)	3,161 (18%)	3,045 (15%)	6,206 (17%)
*Cardiovascular medication use*						
AARAS, n (%)	1,688 (29%)	1,896 (29%)	3,584 (29%)	5,137 (30%)	5,811 (29%)	10,948 (29%)
Diuretics, n (%)	31 (0.5%)	18 (0.3%)	49 (0.4%)	97 (0.6%)	25 (0.1%)	122 (0.3%)
Xa-inhibitors, n (%)	91 (1.6%)	85 (1.3%)	176 (1.4%)	162 (0.9%)	219 (1.1%)	381 (1.0%)
Vitamin K antagonists, n (%)	18 (0.3%)	17 (0.3%)	35 (0.3%)	26 (0.2%)	53 (0.3%)	79 (0.2%)
Statins, n (%)	1,874 (33%)	2,160 (33%)	4,034 (33%)	5,617 (33%)	6,318 (32%)	11,935 (32%)
Nitrates, n (%)	177 (3.1%)	244 (3.7%)	421 (3.4%)	506 (2.9%)	605 (3.0%)	1,111 (3.0%)
Platelet, n (%)	1,023 (18%)	1,154 (17%)	2,177 (18%)	2,995 (17%)	2,932 (15%)	5,927 (16%)
*Diabetes medication use*						
Insulin, n (%)	230 (4.0%)	205 (3.1%)	435 (3.5%)	486 (2.8%)	436 (2.2%)	922 (2.5%)
GLP1-RA, n (%)	0 (0%)	< 5 (< 0.1%)	< 5 (< 0.1%)	< 5 (< 0.1%)	< 5 (< 0.1%)	< 5 (< 0.1%)
SGLT2-i, n (%)	< 5 (< 0.1%)	6 (< 0.1%)	7 (< 0.1%)	0 (0%)	< 5 (< 0.1%)	< 5 (< 0.1%)
Biguanides, n (%)	2,381 (41%)	2,570 (39%)	4,951 (40%)	6,747 (39%)	7,337 (37%)	14,084 (38%)
*Osteoporosis medication use*						
Bisphosphonate, n (%)	109 (1.9%)	422 (6.4%)	531 (4.3%)	166 (1.0%)	684 (3.4%)	850 (2.3%)
Denosumab, n (%)	< 5 (< 0.1%)	36 (0.5%)	40 (0.3%)	< 5 (< 0.1%)	59 (0.3%)	63 (0.2%)

Data is reported as percentages or as mean/median values, with variability expressed as either SD or IQR, depending on data distribution

AMI, acute myocardial infarction; CKD, chronic kidney disease; AFib, atrial fibrillation; AFL, atrial flutter; AARAS, agents acting on the renin–angiotensin system; GLP1-RA, glucagon-like peptide-1 receptor agonist; MOF, Major osteoporotic fracture; SGLT2-i, sodium–glucose cotransporter-2 inhibitor; Composite CVD exposure, includes atherosclerosis; AMI; stroke; hypertension; CKD; AFib/AFL; heart failure; and hypercholesterolemia. Clinical CVD, AMI; stroke; heart failure; AFib/AFL and atherosclerosis. Subclinical CVD, Hypercholesterolemia; hypertension and CKD

In this table, diabetic nephropathy and CKD are presented separately for coding-related reasons only. The CKD category also encompasses diabetic nephropathy. Any prior fracture includes fractures recorded up to but excluding the index date

### Conditional Logistic Regression

#### CVD Grouping

A significant association was observed between composite CVD and fracture odds when any fracture was used as the primary outcome (OR 1.20, 95% CI 1.15–1.26; p < 0.01). Sex-stratified analyses showed similar effect estimates for men (OR 1.16, 95% CI 1.08–1.24; p < 0.01) and women (OR 1.24, 95% CI 1.16–1.31; p < 0.01), with no significant difference between sexes (z = –1.37; p = 0.17).

**Fig. 2:**
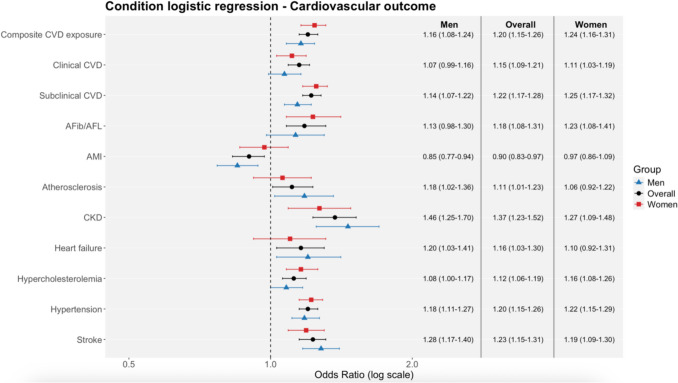
Conditional logistic regression analysis presenting the association between CVD outcome and fracture risk, stratified by sex. Results are displayed as OR with 95% CI on a logarithmic scale. Composite CVD exposure: Atherosclerosis, AMI, stroke, hypertension, CKD, AFib/AFL, heart failure, and hypercholesterolemia. Clinical CVD: AMI, stroke, heart failure, AFib/AFL and atherosclerosis Subclinical CVD: Hypercholesterolemia, hypertension and CKD. AMI, acute myocardial infarction; CKD, chronic kidney disease; AFib, atrial fibrillation; AFL, atrial flutter.

Both clinical and subclinical CVD were associated with increased fracture odds. Clinical CVD was associated with higher odds overall (OR 1.15, 95% CI 1.09–1.21; p < 0.01), reaching significance in women (OR 1.11, 95% CI 1.03–1.19; p < 0.01) but not in men (OR 1.07, 95% CI 0.99–1.16; p = 0.11), with no sex difference (z = –0.65; p = 0.52). Subclinical CVD showed the strongest association (OR 1.22, 95% CI 1.17–1.28; p < 0.01), significant in both men (OR 1.14, 95% CI 1.07–1.22; p < 0.01) and women (OR 1.25, 95% CI 1.17–1.32; p < 0.01), with a borderline sex difference (z = –1.86; p = 0.06). Full numerical estimates are presented in Fig. 2 and supplementary section S3 table A1.

In contrast, when MOF was examined as the secondary outcome, associations were attenuated and no longer significant for composite CVD (OR 0.90, 95% CI 0.76–1.07; p = 0.24), clinical CVD (OR 0.95, 95% CI 0.79–1.15; p = 0.59), or subclinical CVD (OR 0.90, 95% CI 0.76–1.06; p = 0.20), with no evidence of sex-specific differences. Full numerical estimates are presented in supplementary section S3 table A2.

#### CVD Subtypes

When any fracture was used as the primary outcome, most CVD subtypes were significantly associated with increased fracture odds, including AFib/AFL (OR 1.18, 95% CI 1.08–1.31; p < 0.01), atherosclerosis (OR 1.11, 95% CI 1.01–1.23; p = 0.03), CKD (OR 1.37, 95% CI 1.23–1.52; p < 0.01), heart failure (OR 1.16, 95% CI 1.03–1.30; p = 0.01), hypercholesterolemia (OR 1.12, 95% CI 1.06–1.19; p < 0.01), hypertension (OR 1.20, 95% CI 1.15–1.26; p < 0.01), and stroke (OR 1.23, 95% CI 1.15–1.31; p < 0.01). In contrast, AMI was inversely associated with fracture odds (OR 0.90, 95% CI 0.83–0.97; p < 0.01). Several associations showed sex-specific significance, although no statistically significant sex differences were observed overall. Full numerical estimates are presented in Fig. 2 and supplementary section S3 table A1.

When MOF was examined as the secondary outcome, associations were largely attenuated and no longer statistically significant for most CVD subtypes, including AFib/AFL, AMI, CKD, heart failure, hypercholesterolemia, hypertension, and stroke (all p > 0.05). An exception was atherosclerosis, which showed a reduced odds of MOF overall (OR 0.67, 95% CI 0.48–0.94; p = 0.02), although sex-stratified estimates were non-significant. No significant sex differences were observed for any CVD subtype. Full numerical estimates are presented in supplementary section S3 Table A2.

#### CVD Medication

**Fig. 3:**
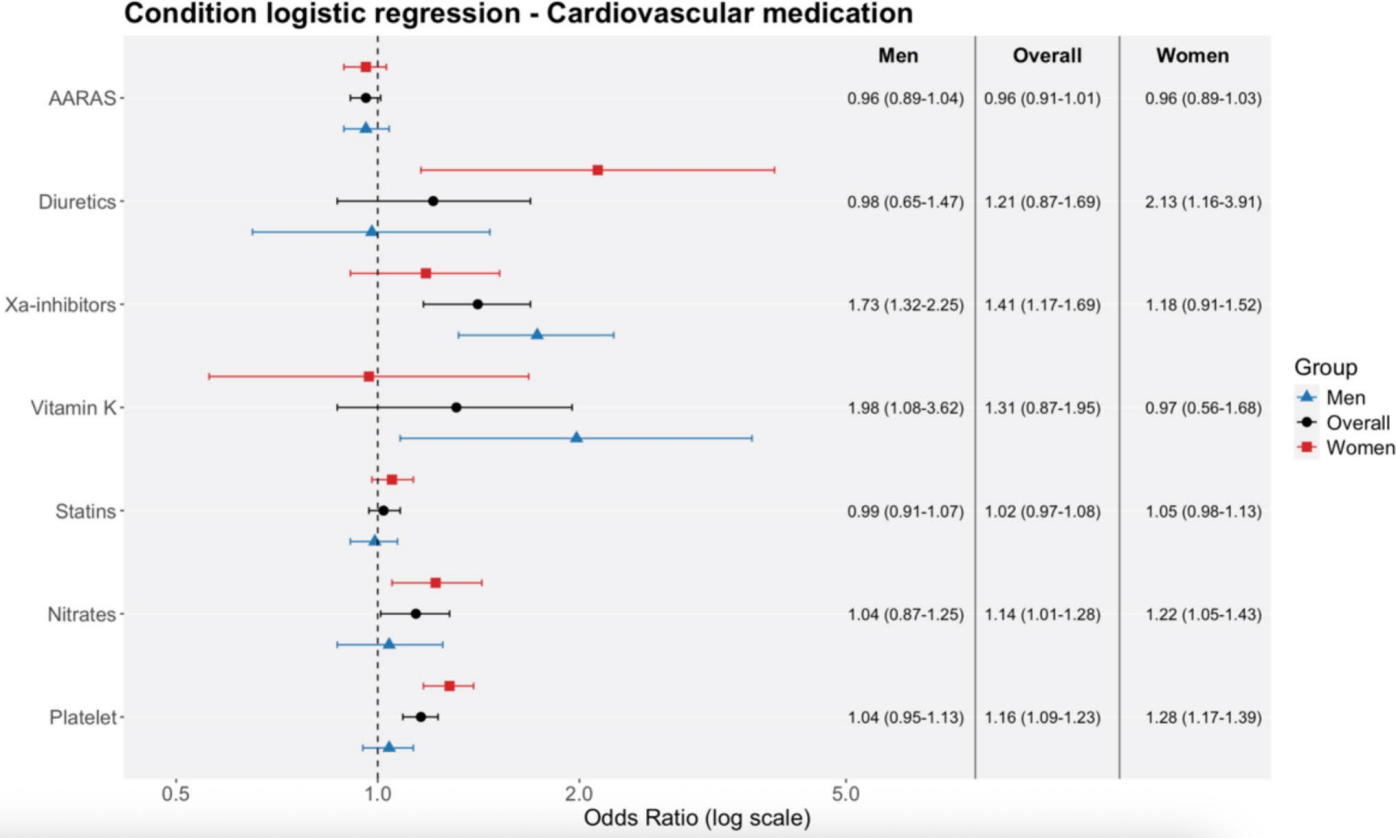
Conditional logistic regression analysis presenting the association between cardiovascular medication and fracture risk, stratified by sex. Results are displayed as OR with 95% CI on a logarithmic scale. AARAS, agents acting on the renin–angiotensin system.

When any fracture was used as the primary outcome, increased fracture odds were observed with Xa-inhibitors (OR 1.41, 95% CI 1.17–1.69; p < 0.01), nitrates (OR 1.14, 95% CI 1.01–1.28; p = 0.03), and antiplatelet agents (OR 1.16, 95% CI 1.09–1.23; p < 0.01), while no associations were seen for statins or antiarrhythmic agents. Sex-specific differences were observed for diuretics (men: OR 0.98, 95% CI 0.65–1.47; p = 0.92; women: OR 2.13, 95% CI 1.16–3.91; p = 0.01; z = –2.09; p = 0.04), Xa-inhibitors (men: OR 1.73, 95% CI 1.32–2.25; p < 0.01; women: OR 1.18, 95% CI 0.91–1.52; p = 0.21; z = 2.03; p = 0.04), and antiplatelet agents (men: OR 1.04, 95% CI 0.95–1.13; p = 0.46; women: OR 1.28, 95% CI 1.17–1.39; p < 0.01; z = –3.29; p < 0.01). Full numerical estimates are presented in Fig. 3 and Supplementary section S3 table A1.

When MOF was examined as the secondary outcome, no statistically significant associations were observed for any cardiovascular medication class, including Xa-inhibitors (OR 0.82, 95% CI 0.41–1.63), diuretics (OR 1.76, 95% CI 0.41–7.62), nitrates (OR 0.80, 95% CI 0.51–1.27), antiplatelet agents (OR 1.04, 95% CI 0.81–1.32), statins (OR 1.01, 95% CI 0.82–1.26), vitamin K antagonists (OR 0.44, 95% CI 0.10–1.91), or antiarrhythmic agents (OR 0.99, 95% CI 0.79–1.24), with no evidence of sex-specific differences. Full numerical estimates are presented in supplementary section S3 table A2.

## Discussion

In this nationwide matched case–control study of people with T2D, CVD recorded after T2D diagnosis and before the fracture index date was associated with approximately 20% higher odds of any fracture. This association was observed for composite CVD and for both clinical and subclinical CVD. Several individual CVD phenotypes, including AFib/AFL, atherosclerosis, CKD, heart failure, hypercholesterolemia, hypertension, and stroke, were associated with higher odds of any fracture, whereas AMI showed a modest inverse association. Selected cardiovascular medications, including factor Xa inhibitors, nitrates, and antiplatelet agents, were also associated with higher fracture odds. Overall, the main finding is that cardiovascular and cardiometabolic disease burden may be a clinically relevant marker of broader fracture vulnerability in people with T2D.

When MOF was examined as the secondary outcome, associations were attenuated and no longer statistically significant for composite CVD, clinical CVD, subclinical CVD, most individual cardiovascular phenotypes, and cardiovascular medication classes. This attenuation should be interpreted cautiously. First, the number of MOF events was lower than the number of any fracture events, reducing statistical power and widening confidence intervals. Second, any fracture and MOF may capture partly different fracture phenotypes. Any fracture includes a broader spectrum of fracture sites and mechanisms, whereas MOF is more closely linked to classical osteoporotic fracture risk. Therefore, the weaker associations for MOF may reflect reduced power, differences in fracture phenotype, or both.

Fall-related mechanisms are biologically plausible in the association between CVD and fracture, but they cannot be directly inferred from the present data. Cardiovascular disease may contribute to fall risk through syncope, arrhythmia-related instability, orthostatic hypotension, reduced exercise tolerance, and frailty [11]. Cardiovascular medications may also increase fall risk, particularly through effects on blood pressure, rhythm, and postural stability [12]. In people with diabetes, neuropathy, impaired postural control, and balance dysfunction may further increase susceptibility to falls and fall-related injuries [13, 14]. Moreover, previous evidence suggests that people with T2D and CVD have increased fall risk, supporting the biological plausibility of this pathway [15]. However, most fractures, including many MOF events, may be precipitated by falls, and the lack of association for MOF should not be interpreted as evidence that the association between CVD and any fracture is mediated by falls. Because fall history, trauma mechanism, physical function, and frailty were not directly measured, the potential contribution of fall-related mechanisms remains hypothesis-generating.

### Cardiovascular Disease

Composite CVD exposure was associated with higher odds of any fracture among people with T2D, and this association did not differ substantially by sex. This aligns with evidence linking cardiovascular dysfunction and cardiovascular risk factor burden with fracture risk, including studies showing that cardiovascular biomarkers and adverse cardiovascular health profiles are associated with incident fracture risk [28, 29]. Rather than suggesting a direct causal skeletal effect of CVD, these findings may indicate that cardiovascular morbidity captures a broader vulnerability phenotype characterized by multimorbidity, vascular dysfunction, impaired physical function, frailty, and higher fall susceptibility.

Separating clinical CVD from operationally defined subclinical CVD provided additional insight. Both groupings were associated with higher odds of any fracture, but the estimate appeared stronger for subclinical CVD. This finding should be interpreted with caution. One possible explanation is that individuals with subclinical CVD may have longer survival and therefore more time at risk for fracture, whereas individuals with established clinical CVD may have a higher competing risk of death before sustaining or registering a fracture. Competing mortality may therefore attenuate observed fracture associations among people with more severe clinical cardiovascular disease. In addition, subclinical CVD in this study included common conditions such as hypertension, hypercholesterolemia, and CKD, which were more prevalent than several clinical CVD subtypes and therefore provided greater statistical power to detect associations.

Most individual CVD subtypes were associated with higher odds of any fracture, supporting the overall finding that cardiovascular morbidity is linked to fracture vulnerability in T2D. These associations are compatible with several shared pathways. Inflammation is central to atherosclerosis and may also contribute to bone loss and skeletal fragility [30]. Coronary and systemic microvascular dysfunction may reflect impaired tissue perfusion and broader vascular disease burden [31]. Autonomic imbalance may link cardiovascular dysfunction with orthostatic instability and fall susceptibility [32]. In addition, frailty and reduced physical function may increase both cardiovascular vulnerability and fracture risk [33]. However, interpretation of subtype-specific findings should account for differences in the number of exposed individuals and statistical precision. Common conditions such as hypertension and hypercholesterolemia provided more statistical power than less frequent exposures such as AFib/AFL, heart failure, CKD, or atherosclerosis. Therefore, differences in statistical significance across CVD subtypes may reflect both true differences in association and differences in exposure prevalence and precision.

Specific CVD subtypes should be interpreted cautiously. CKD was associated with higher fracture odds, consistent with known disturbances in mineral metabolism and bone turnover in kidney disease [34, 35]. Hypertension showed increased fracture odds, in line with previous evidence linking hypertension with fracture risk [36]. Hypercholesterolemia was also associated with higher fracture odds, consistent with prospective evidence linking serum lipid traits with fracture and osteoporosis risk [37]. Stroke is a well-recognized contributor to fracture risk through impaired balance, neuromuscular deficits, reduced mobility, and falls [38, 39]. The inverse association observed for AMI was unexpected and should not be interpreted as protective. Previous studies have reported increased fracture risk after myocardial infarction [40], and the inverse association in our study may instead reflect survivor bias, competing mortality, more intensive post-AMI clinical follow-up, or partial overmatching because weighted CCI includes AMI [41, 42].

### Cardiovascular Medication

Selected cardiovascular medications, including factor Xa inhibitors, nitrates, and antiplatelet agents, were associated with higher odds of any fracture. Cardiovascular drugs have previously been linked to fall-related outcomes, supporting the plausibility that medication use may mark increased fall vulnerability [12]. Anticoagulant use has also been associated with fracture risk in nationwide registry data [43]. However, these associations should primarily be interpreted as markers of underlying cardiovascular disease severity, multimorbidity, polypharmacy, and frailty rather than evidence of direct pharmacological effects on bone. Confounding by indication is likely, as treatment use may reflect more advanced cardiovascular disease and reduced physiological reserve.

As with CVD subtype analyses, interpretation of medication-specific findings should consider exposure prevalence and statistical power. Medication classes with few exposed individuals had wider confidence intervals and lower power to detect associations, especially in the MOF analyses. The potential role of statin therapy remains of interest, as some studies have suggested a protective effect against fractures, although we found no such association in our data [44]. Sex-specific patterns were observed for selected medication classes, including diuretics, factor Xa inhibitors, and antiplatelet agents. However, these findings should be interpreted cautiously given the number of comparisons, differences in exposure prevalence, and limited precision in some strata.

### Implications for Current Care Models and Prevention

Identification of CVD comorbidity in people with T2D may serve as a pragmatic clinical marker for broader fracture vulnerability. This does not imply that CVD should necessarily be incorporated into osteoporosis prediction tools such as FRAX, particularly because associations were attenuated for MOF. Rather, cardiovascular status may help identify people with T2D who could benefit from broader fracture prevention strategies, including fall-risk assessment, review of medication burden, and evaluation of skeletal health. However, such implications should be considered cautiously, as the present observational registry-based study cannot establish causal mechanisms.

### Strengths and Limitation

This nationwide registry-based matched case–control study benefits from complete population coverage and precise individual-level linkage across Danish health registries. The 1:3 matching on age, sex, T2D onset date, and weighted CCI improved comparability between cases and controls while reducing confounding by overall comorbidity burden. Fracture status was analysed using conditional logistic regression, and medication exposure was based on redeemed prescriptions with a minimum one-year history to increase the likelihood of sustained treatment.

Several limitations should however be considered. Matching included weighted CCI, which incorporates cardiovascular conditions and may have resulted in partial overmatching, potentially attenuating associations between CVD and fracture. However, specific CVD subtypes were analysed separately, and any attenuation would likely bias results towards the null. Because people with CVD recorded before their first documented T2D diagnosis were excluded, the findings may not be generalizable to people with pre-existing cardiovascular disease before T2D onset. This restriction was applied to ensure a consistent temporal sequence in which T2D preceded CVD exposure.

The study relied on ICD and ATC codes from nationwide registers. Therefore, individual events could not be adjudicated, diagnoses could not be clinically standardized beyond registry coding, and misclassification of CVD exposures, comorbidities, medication exposure, or fracture outcomes cannot be excluded. Clinically silent or radiographically detected vertebral fractures not registered as clinical diagnoses would not be captured. Although Danish health registries provide high-quality nationwide data, reliance on administrative codes remains an inherent limitation of the study design.

Fracture coding did not permit differentiation between low- and high-trauma events, and misclassification of fracture site cannot be excluded. The lack of information on trauma severity and fall history limits interpretation of whether observed associations reflect skeletal fragility, fall-related mechanisms, or both. The absence of significant associations for MOF may partly reflect the lower number of MOF events and reduced statistical power in these secondary analyses. Furthermore, because fall history, trauma severity, physical function, and frailty were not directly measured, the present data cannot determine whether observed associations are mediated by fall-related pathways, skeletal fragility, or both. We did not perform separate analyses of non-MOF fractures, as this was not a predefined outcome; however, such analyses may provide further insight into whether associations for any fracture are driven by fractures outside the MOF category.

Medication analyses are susceptible to confounding by indication, as dispensing patterns reflect disease severity and frailty. Polypharmacy, cumulative treatment burden, dose–response relationships, and detailed duration of therapy were not assessed. Although exposure required ≥ 2 prescriptions within 365 days to reduce misclassification during the initiation phase, redeemed prescriptions do not guarantee adherence, and some exposure misclassification is inevitable. Such misclassification is likely non-differential and may bias associations towards the null.

Important clinical and lifestyle confounders were unavailable, including body mass index, smoking, alcohol use, physical activity, bone mineral density, vitamin D status, HbA1c, and validated frailty measures. Residual confounding is therefore possible, and observed associations may partly reflect unmeasured health status rather than direct biological mechanisms. In addition, competing mortality may have influenced the observed associations, particularly among people with more severe clinical CVD, who may have been less likely to survive long enough to sustain or register a fracture.

The study period (2013–2021) encompassed evolving diabetes management, including increasing use of GLP1 receptor agonists and SGLT2 inhibitors. Uptake was low in this cohort, likely reflecting both calendar time effects and relatively short diabetes duration at index, as treatment intensification typically occurs stepwise. Calendar year was not included as a separate covariate, and residual temporal effects cannot be excluded. The short diabetes duration limits generalisability to people with longer-standing T2D, in whom fracture risk may be greater.

Hypertension, hypercholesterolaemia, and CKD were operationally grouped as subclinical CVD within a vascular continuum framework. We acknowledge that these conditions may also be considered cardiovascular risk factors or cardiometabolic–renal manifestations rather than manifest clinical CVD, and the term subclinical CVD should therefore be interpreted in this operational sense. Finally, multiple cardiovascular phenotypes and medication classes were examined without formal multiplicity correction; therefore, some findings may represent type I error. Additionally, results reflect Danish healthcare structures and coding practices and may not fully generalise to other settings.

## Conclusion

Among people with T2D without recorded CVD before diabetes diagnosis, subsequent CVD was associated with higher odds of fracture when any fracture was used as the primary outcome, with similar associations in women and men. Composite, clinical and subclinical CVD, as well as several CVD subtypes, was associated with increased odds, whereas AMI showed a modest inverse association. Selected cardiovascular medications were associated with higher odds for any fracture, with limited sex-specific effects. However, when MOF was examined as the secondary outcome, associations were attenuated and largely non-significant, which may partly reflect lower statistical power and/or differences in the mechanisms underlying any fracture versus MOF. Overall, these findings highlight the close interrelation between cardiovascular and skeletal health in T2D and support integrated, sex-informed strategies for fracture risk assessment and prevention.

## Supplementary Information

Below is the link to the electronic supplementary material.

Supplementary file1 (DOCX 45 KB)

## Data Availability

The data are not available for sharing, as they contain personally identifiable information. Only the anonymized and published data in this study are available for sharing.

## Abbreviations

AFib: Atrial fibrillation
AFL: Atrial flutter
AMI: Acute myocardial infarction
ATC: Anatomical Therapeutic Chemical
CCI: Charlson Comorbidity Index
CI: Confidence intervals
CKD: Chronic kidney disease
CPR: Civil personal registration
CVD: Cardiovascular diseases
GLP1-RA: Glucagon-like peptide-1 receptor agonist
MOF: Major osteoporotic fractures
IQR: Interquartile range
OR: Odds ratios
SD: Standard deviation
SGLT2-i: Sodium–glucose cotransporter-2 inhibitor
T2D: Type 2 diabetes mellitus
PCOS: Polycystic ovary syndrome
