# Supplementary material for: Cardiovascular Disease and Fracture Risk in People with Type 2 Diabetes: A Nationwide Matched Case–Control Study
Source: Calcif Tissue Int. 2026 May 29;117(1):93. doi: 10.1007/s00223-026-01556-0 (PMC13219164; doi:10.1007/s00223-026-01556-0)
Supplement: Supplementary file 1 — Supplementary file1 (DOCX 45 KB) [file 223_2026_1556_MOESM1_ESM.docx]

Supplementary materials

S1: Table 1, using MOF outcome for cases instead of any fracture

| **Variable** | **People with T2D and MOF fractures (Cases)** | | | **People with T2D without MOF fractures (Controls)** | | |
| --- | --- | --- | --- | --- | --- | --- |
|  | **Men**  **(n = 2,752)** | **Women**  **(n = 4,509)** | **Overall**  **(n = 7,261)** | **Men**  **(n = 20,272)** | **Women**  **(n = 22,027)** | **Overall**  **(n = 42,299)** |
| Age, median [IQR] | 70.27 [59.59;79.01] | 75.37 [66.58;83.14] | 73.59 [63.62;81.93] | 64.46 [54.77;75.17] | 70.35 [59.95;78.61] | 68.22 [57.27;77.22] |
| CCI-weighted, median [IQR] | 1.00 [0.00;2.00] | 1.00 [0.00;2.00] | 1.00 [0.00;2.00] | 1.00 [0.00;2.00] | 1.00 [0.00;2.00] | 1.00 [0.00;2.00] |
| Any prior fracture, n (%) | 1,409 (51%) | 2,601 (58%) | 4,010 (55%) | 6,191 (31%) | 6,524 (30%) | 12,715 (30%) |
| **Diabetes status** | | | | | | |
| Diabetes duration, years, median [IQR] | 2.04 [0.79;3.82] | 2.03 [0.78;3.76] | 2.04 [0.79;3.78] | 2.26 [0.95;4.08] | 2.41 [1.02;4.23] | 2.34 [0.98;4.16] |
| Nephropathy, n (%) | 91 (3.3%) | 90 (2.0%) | 181 (2.5%) | 509 (2.5%) | 392 (1.8%) | 901 (2.1%) |
| Neuropathy, n (%) | 383 (14%) | 595 (13%) | 978 (13%) | 2,128 (10%) | 2,803 (13%) | 4,931 (12%) |
| Retinopathy, n (%) | 103 (3.7%) | 158 (3.5%) | 261 (3.6%) | 629 (3.1%) | 653 (3.0%) | 1,282 (3.0%) |
| **Cardiovascular status** | | | | | | |
| Composite CVD exposure, n (%) | 1,545 (56%) | 2,604 (58%) | 4,149 (57%) | 9,790 (48%) | 11,029 (50%) | 20,819 (49%) |
| Clinical CVD, n (%) | 987 (36%) | 1,268 (28%) | 2,255 (31%) | 5,649 (28%) | 4,993 (23%) | 10,642 (25%) |
| Subclinical CVD, n (%) | 1,336 (49%) | 2,349 (52%) | 3,685 (51%) | 8,275 (41%) | 9,895 (45%) | 18,170 (43%) |
| Atherosclerosis, n (%) | 182 (6.6%) | 215 (4.8%) | 397 (5.5%) | 873 (4.3%) | 892 (4.0%) | 1,765 (4.2%) |
| AMI, n (%) | 314 (11%) | 324 (7.2%) | 638 (8.8%) | 2,383 (12%) | 1,337 (6.1%) | 3,720 (8.8%) |
| Stroke, n (%) | 527 (19%) | 671 (15%) | 1,198 (16%) | 2,414 (12%) | 2,562 (12%) | 4,976 (12%) |
| Hypertension, n (%) | 1,155 (42%) | 2,127 (47%) | 3,282 (45%) | 7,070 (35%) | 8,995 (41%) | 16,065 (38%) |
| CKD, n (%) | 168 (6.1%) | 173 (3.8%) | 341 (4.7%) | 674 (3.3%) | 657 (3.0%) | 1,331 (3.1%) |
| AFib/AFL, n (%) | 140 (5.1%) | 250 (5.5%) | 390 (5.4%) | 916 (4.5%) | 869 (3.9%) | 1,785 (4.2%) |
| Heart failure, n (%) | 154 (5.6%) | 137 (3.0%) | 291 (4.0%) | 742 (3.7%) | 521 (2.4%) | 1,263 (3.0%) |
| Hypercholesterolemia, n (%) | 578 (21%) | 774 (17%) | 1,352 (19%) | 3,705 (18%) | 3,428 (16%) | 7,133 (17%) |
| **Cardiovascular medication use** | | | | | | |
| AARAS, n (%) | 796 (29%) | 1,281 (28%) | 2,077 (29%) | 6,029 (30%) | 6,426 (29%) | 12,455 (29%) |
| Diuretics, n (%) | 12 (0.4%) | 12 (0.3%) | 24 (0.3%) | 116 (0.6%) | 31 (0.1%) | 147 (2.7%) |
| Xa-inhibitors, n (%) | 50 (1.8%) | 63 (1.4%) | 113 (1.6%) | 203 (1.0%) | 241 (1.1%) | 444 (1.0%) |
| Vitamin K antagonists, n (%) | 5 (0.2%) | 11 (0.2%) | 16 (0.2%) | 39 (0.2%) | 59 (0.3%) | 98 (0.2%) |
| Statins, n (%) | 852 (31%) | 1,428 (32%) | 2,280 (31%) | 6,639 (33%) | 7,050 (32%) | 13,689 (32%) |
| Nitrates, n (%) | 104 (3.8%) | 184 (4.1%) | 288 (4.0%) | 579 (2.9%) | 665 (3.0%) | 1,244 (2.9%) |
| Platelet, n (%) | 517 (19%) | 820 (18%) | 1,337 (18%) | 3,501 (17%) | 3,266 (15%) | 6,767 (16%) |
| **Diabetes medication use** | | | | | | |
| Insulin, n (%) | 112 (4.1%) | 121 (2.7%) | 233 (3.2%) | 604 (3.0%) | 520 (2.4%) | 1,124 (2.7%) |
| GLP1-RA, n (%) | 0 (0%) | 0 (0%) | 0 (0%) | <5 (<0.1%) | <5 (<0.1%) | <5 (<0.1%) |
| SGLT2-i, n (%) | 0 (0%) | <5 (<0.1%) | <5 (<0.1%) | <5 (<0.1%) | 6 (<0.1%) | 7 (<0.1%) |
| Biguanides, n (%) | 1,055 (38%) | 1,663 (37%) | 2,718 (37%) | 8,073 (40%) | 8,244 (37%) | 16,317 (39%) |
| **Osteoporosis medication use** | | | | | | |
| Bisphosphonate, n (%) | 61 (2.2%) | 309 (6.9%) | 370 (5.1%) | 214 (1.1%) | 797 (3.6%) | 1,011 (2.4%) |
| Denosumab, n (%) | <5 (<0.1%) | 24 (0.5%) | 25 (0.3%) | 7 (<0.1%) | 71 (0.3%) | 78 (0.2%) |

Data is reported as percentages or as mean/median values, with variability expressed as either SD or IQR, depending on data distribution.

Abbreviations: AMI = acute myocardial infarction, CKD = chronic kidney disease, AFib = atrial fibrillation, AFL = atrial flutter, AARAS = agents acting on the renin–angiotensin system, GLP1-RA = glucagon-like peptide-1 receptor agonist, MOF = Major osteoporotic fracture, SGLT2-i = sodium–glucose cotransporter-2 inhibitor, Composite CVD exposure = includes atherosclerosis, AMI, stroke, hypertension, CKD, AFib/AFL, heart failure, and hypercholesterolemia. Clinical CVD = AMI, stroke, heart failure, AFib/AFL and atherosclerosis. Subclinical CVD = Hypercholesterolemia, hypertension and CKD.

Note: In this table, diabetic nephropathy and CKD are presented separately for coding-related reasons only. The CKD category also encompasses diabetic nephropathy. Any prior fracture includes fractures recorded up to but excluding the index date.

S2: ICD-8, ICD-10 and ATC codes

Diseases and medication:

| Diabetes complications | ICD 10 codes |
| --- | --- |
| Retinopathy | H35.0, E10.3, E11.3, E12.3, E13.3, E14.3, H31.0, H35.1, H36.0, H36.8, H35.2, H35.7, H33.4, I70.8, H35.5 |
| Nephropathy | E85.0, N08, N07, E10.2, E11.2, E12.2, E13.2, E14.2, N28.9, I12.0, I12.9, N14.0, N14.3, N14.4, N15.0, |
| Neuropathy | G56, G57, G58, G60, G62, G61, G63.2, G63.8, G59, G90, M14.6, M49.4, E10.4, E11.4, E12.4, E13.4, E14.4 |
| Atherosclerosis | I70 |
| Cardiovascular diseases | ICD 10 – codes |
| AMI | I21 |
| Stroke | I63, I64, I69, I61.9, R58, I74 (I60, I61) |
| Heart failure | I50.0 + I50.2 + I50.4 |
| Hypercholesterolemia | E78.0-E78.5 |
| Hypertension | I10-I15 |
| CKD | N18 |
| Afli/afla | I148 + I49 + I47.0 + F45.3 |
| Diabetes medication | ATC - codes |
| Biguanides | A10BA |
| Thiazolidinediones (Glitazones) | **A10BC** |
| Sodium-glucose co-transporter 2 (SGLT-2) inhibitors | **A10BF** |
| Glucagon-like peptide-1 (GLP-1) receptor agonists | A10BG |
| Insulins and analogues | A10A |
| Anti-hypertension/ Anti-arythmic medication | ATC - codes |
| Agents Acting on the Renin-Angiotensin System | C09 |
| Adrenergic beta-antagonists (Beta-blockers) | **C02B** |
| Calcium channel blockers | **C02D** |
| Diuretics | **C02E +** C03C |
| Anti-coagulation medication | ATC - codes |
| Direct factor Xa inhibitors | **B01AE** |
| Vitamin K antagonists | **B01AB** |
| Platelet function inhibiting agent | B01AC06 + B01AC04 |
| Cholesterol lowering medication | **ATC-codes** |
| **Statins** | C10AA |
| Osteoporotic medication | **ATC-codes** |
| Bisphosphonate | M05BA |
| Denosumab | M05BX04 |
| Other medication | **ATC-codes** |
| Nitrates | C01DA |

Fracture codes:

| Fracture site (group) | ICD-10 | ICD-8 |
| --- | --- | --- |
| Head | S02, S07 | 800-804 |
| Vertebral | S12, S220, S221, S320, T08, M484, M485 | 805, 806 |
| Ribs | S222-225, S228, S229 | 807 |
| Pelvis | S321-325, S327, S328 | 808 |
| Clavicle | S420 | 810 |
| Scapula | S421 | 811 |
| Humerus | S422-424 | 812 |
| Forearm | S52 | 813 |
| Hand | S620-927 | 814-816 |
| Hip | S720-722 | 820 |
| Femur | S723-4, S727-729 | 821 |
| Patella | S820 | 822 |
| Lower leg | S821-824, S827 | 823 |
| Ankle | S825, S826, S828, S829 | 824 |
| Foot | S920-925, S927, S929 | 825 |
| MOF | Vertebral + Humerus + Forearm + Hip | |

Charlson Comorbidity Index:

| CCI disease group | ICD-10 | ICD-8 | Weighting |
| --- | --- | --- | --- |
| Any malignancy, including leukemia and lymphoma, but does not include non-melanoma skin cancer | C00-C86, C88-C97 | 140-172, 174, 180-207, 209 | 2 |
| Cerebrovascular | G45, I60-64, I67, I69 | 430-438 | 1 |
| Chronic obstructive pulmonary disease (COPD) | J43, J44 | 491, 492 | 1 |
| Congestive heart failure (CHF) | I110, I130, I132, I255, I420, I426, I427,  I428, I429, I43, I50 | 42508, 42509, 4270, 4271, 428 | 1 |
| Dementia | F00-03, F051, G30, G311, G319 | 290 | 1 |
| Diabetes | E100, E101, E110, E111, E120, E121, E130, E131, E140, E141 | 25000, 25007, 25008 | 1 |
| Diabetes with end organ damage | E102-105, E107, E112-117, E122-127, E132-137, E142-147 | 25001, 25002, 25003, 25004, 25005 | 2 |
| Hemiplegia, tetraplegia | G114, G80-82, G830-833, G838 | 343, 344 | 2 |
| Aids | B20-24, F024, O987,  R75, Z114, Z219, Z711 | NA | 6 |
| Metastatic cancer | C77-80 | 196-199 | 6 |
| Mild liver disease | B15-19, K703, K73, K746, K703, K754 | 070, 571, 573 | 1 |
| Moderate or severe kidney disease | N032-037, N052-057, N11, N18, N19, N250, I120, I131, Q611-614, Z49, Z940, Z992 | 582, 583, 584, 792, 5930, 40399, 40499, 79299, Y2901 | 2 |
| Moderate or severe liver disease | R18 (+Any code for mild liver disease in combination with ascites) I850  I859  I982  I983 | 7853 (+Any code for mild liver disease in combination with ascites) 4560  5719  57302 | 3 |
| Myocardial infarction | I21  I22  I252 | 410  411  41201  41291 | 1 |
| Other chronic pulmonary disease | J41, J42, J45, J46, J47,  J60-70 | 490, 493, 515-518 | 1 |
| Peripheral vascular disease | I70, I71, I731, I738, I739, I771, I790, I792, K55 | 440, 441, 4431, 4439 | 1 |
| Rheumatic | M05, M06, M123, M070–073, M08, M13, M30, M313-316, M32, M33, M34, M350, M351, M353, M45-46 | 446, 69600, 7120–7123, 7125, 716, 7340, 7341, 7349 | 1 |
| (Peptic) Ulcer disease | K25-K28 | 531-534 | 1 |

S3: Conditional logistic regression

Conditional logistic regression models with any fracture as the outcome

|  | **Overall** | | | **Men** | | | **Women** | | | **Between sexes** | |
| --- | --- | --- | --- | --- | --- | --- | --- | --- | --- | --- | --- |
| **Variable** | **OR** | **CI** | **p-value** | **OR** | **CI** | **p-value** | **OR** | **CI** | **p-value** | **z-value** | **p-value** |
| **Cardiovascular outcome** | | | | | | | | | | | |
| Composite CVD exposure | 1.20 | 1.15-  1.26 | <0.01* | 1.16 | 1.08-1.24 | <0.01* | 1.24 | 1.16-1.31 | <0.01* | -1.37 | 0.17 |
| Clinical CVD | 1.15 | 1.09-  1.21 | <0.01* | 1.07 | 0.99-1.16 | 0.11 | 1.11 | 1.03-1.19 | <0.01* | -0.65 | 0.52 |
| Subclinical CVD | 1.22 | 1.17-  1.28 | <0.01* | 1.14 | 1.07-1.22 | <0.01* | 1.25 | 1.17-1.32 | <0.01* | -1.86 | 0.06 |
| AFib/AFL | 1.18 | 1.08-  1.31 | <0.01* | 1.13 | 0.98-1.30 | 0.10 | 1.23 | 1.08-1.41 | <0.01* | -0.93 | 0.35 |
| AMI | 0.90 | 0.83-  0.97 | <0.01* | 0.85 | 0.77-0.94 | <0.01* | 0.97 | 0.86-1.09 | 0.61 | -1.61 | 0.11 |
| Atherosclerosis | 1.11 | 1.01-  1.23 | 0.03* | 1.18 | 1.02-1.36 | 0.03* | 1.06 | 0.92-1.22 | 0.44 | 1.06 | 0.29 |
| CKD | 1.37 | 1.23-  1.52 | <0.01* | 1.46 | 1.25-1.70 | <0.01* | 1.27 | 1.09-1.48 | <0.01* | 1.27 | 0.21 |
| Heart failure | 1.16 | 1.03-  1.30 | 0.01* | 1.20 | 1.03-1.41 | 0.02* | 1.10 | 0.92-1.31 | 0.31 | 0.78 | 0.43 |
| Hypercholesterolemia | 1.12 | 1.06-  1.19 | <0.01* | 1.08 | 1.00-1.17 | 0.07 | 1.16 | 1.08-1.26 | <0.01* | -1.37 | 0.17 |
| Hypertension | 1.20 | 1.15-  1.26 | <0.01* | 1.18 | 1.11-1.27 | <0.01* | 1.22 | 1.15-1.29 | <0.01* | -0.58 | 0.56 |
| Stroke | 1.23 | 1.15-  1.31 | <0.01* | 1.28 | 1.17-1.40 | <0.01* | 1.19 | 1.09-1.30 | <0.01* | 1.12 | 0.26 |
| **Cardiovascular medication** | | | | | | | | | | | |
| AARAS | 0.96 | 0.91-  1.01 | 0.14 | 0.96 | 0.89-1.04 | 0.32 | 0.96 | 0.89-1.03 | 0.25 | 0.04 | 1.00 |
| Diuretics | 1.21 | 0.87-  1.69 | 0.26 | 0.98 | 0.65-1.47 | 0.92 | 2.13 | 1.16-3.91 | 0.01* | -2.09 | 0.04* |
| Xa-inhibitors | 1.41 | 1.17-  1.69 | <0.01* | 1.73 | 1.32-2.25 | <0.01* | 1.18 | 0.91-1.52 | 0.21 | 2.03 | 0.04* |
| Vitamin K | 1.31 | 0.87-  1.95 | 0.19 | 1.98 | 1.08-3.62 | 0.03* | 0.97 | 0.56-1.68 | 0.91 | 1.72 | 0.09 |
| Statins | 1.02 | 0.97-  1.08 | 0.40 | 0.99 | 0.91-1.07 | 0.81 | 1.05 | 0.98-1.13 | 0.20 | -1.04 | 0.30 |
| Nitrates | 1.14 | 1.01-  1.28 | 0.03* | 1.04 | 0.87-1.25 | 0.68 | 1.22 | 1.05-1.43 | 0.01* | -1.33 | 0.18 |
| Platelet | 1.16 | 1.09-  1.23 | <0.01* | 1.04 | 0.95-1.13 | 0.46 | 1.28 | 1.17-1.39 | <0.01* | -3.29 | <0.01* |

Table A1: Presents the results of the conditional logistic regression analysis, displaying odds ratios (OR), confidence intervals (CI), p-values, and z-values.

Abbreviations: AMI = acute myocardial infarction; CKD = chronic kidney disease; AFib = atrial fibrillation; AFL = atrial flutter; AARAS = agents acting on the renin–angiotensin system; CVD exposure = includes atherosclerosis, AMI, stroke, hypertension, CKD, AFib/AFL, heart failure, and hypercholesterolemia.

Notes: * = p-value < 0.05; NA = too little variation or few observations. ´Composite CVD exposure´ consists of = AFib/AFL, AMI, atherosclerosis, CKD, heart failure, hypercholesterolemia, hypertension and stroke. ´Clinical CVD´ consists of = AFib/AFL, AMI, heart failure, atherosclerosis and stroke. ´Subclinical CVD´ consists of = CKD, hypercholesterolemia and hypertension.

Conditional logistic regression models with major osteoporotic fracture (MOF) as the outcome

|  | **Overall** | | | **Men** | | | **Women** | | | **Between sexes** | |
| --- | --- | --- | --- | --- | --- | --- | --- | --- | --- | --- | --- |
| **Variable** | **OR** | **CI** | **p-value** | **OR** | **CI** | **p-value** | **OR** | **CI** | **p-value** | **z-value** | **p-value** |
| **Cardiovascular outcome** | | | | | | | | | | | |
| Composite CVD exposure | 0.90 | 0.76-  1.07 | 0.24 | 1.11 | 0.79-1.55 | 0.56 | 0.88 | 0.71-1.08 | 0.23 | 1.13 | 0.26 |
| Clinical CVD | 0.95 | 0.79-  1.15 | 0.59 | 1.15 | 0.80-1.66 | 0.44 | 0.98 | 0.77-1.24 | 0.86 | 0.74 | 0.46 |
| Subclinical CVD | 0.90 | 0.76-  1.06 | 0.20 | 1.09 | 0.79-1.50 | 0.60 | 0.87 | 0.71-1.06 | 0.16 | 1.19 | 0.23 |
| AFib/AFL | 1.01 | 0.72-  1.43 | 0.94 | 0.81 | 0.41-1.62 | 0.56 | 1.14 | 0.76-1.70 | 0.52 | -0.83 | 0.41 |
| AMI | 1.04 | 0.79-  1.35 | 0.80 | 1.16 | 0.75-1.80 | 0.50 | 1.09 | 0.77-1.55 | 0.61 | 0.21 | 0.84 |
| Atherosclerosis | 0.67 | 0.48-  0.94 | 0.02* | 0.85 | 0.47-1.54 | 0.60 | 0.67 | 0.43-1.02 | 0.06 | 0.66 | 0.51 |
| CKD | 0.99 | 0.70-  1.42 | 0.97 | 1.30 | 0.73-2.30 | 0.37 | 0.85 | 0.53-1.34 | 0.47 | 1.14 | 0.25 |
| Heart failure | 0.82 | 0.55-  1.23 | 0.34 | 0.58 | 0.30-1.11 | 0.10 | 1.21 | 0.70-2.10 | 0.50 | -1.69 | 0.09 |
| Hypercholesterolemia | 0.97 | 0.79-  1.20 | 0.81 | 1.15 | 0.79-1.66 | 0.47 | 0.97 | 0.75-1.26 | 0.84 | 0.71 | 0.48 |
| Hypertension | 0.90 | 0.76-  1.06 | 0.20 | 1.14 | 0.83-1.57 | 0.42 | 0.85 | 0.70-1.04 | 0.11 | 1.52 | 0.13 |
| Stroke | 0.84 | 0.68  -  1.04 | 0.12 | 1.04 | 0.69-1.55 | 0.87 | 0.84 | 0.64-1.09 | 0.19 | 0.87 | 0.39 |
| **Cardiovascular medication** | | | | | | | | | | | |
| AARAS | 0.99 | 0.79-  1.24 | 0.93 | 1.21 | 0.79-1.84 | 0.38 | 0.92 | 0.71-1.20 | 0.54 | 1.06 | 0.29 |
| Diuretics | 1.76 | 0.41-  7.62 | 0.45 | 1.97 | 0.18-22.17 | 0.58 | 1.67 | 0.25-10.98 | 0.60 | 0.11 | 0.91 |
| Xa-inhibitors | 0.82 | 0.41-  1.63 | 0.57 | 0.67 | 0.16-2.90 | 0.60 | 0.85 | 0.39-1.88 | 0.69 | -0.27 | 0.78 |
| Vitamin K | 0.44 | 0.10-  1.91 | 0.27 | 1.00 | 0.06-15.99 | 1.00 | 0.30 | 0.05-1.72 | 0.18 | 0.72 | 0.47 |
| Statins | 1.01 | 0.82-  1.26 | 0.90 | 1.19 | 0.81-1.73 | 0.38 | 0.95 | 0.73-1.25 | 0.73 | 0.92 | 0.36 |
| Nitrates | 0.80 | 0.51-  1.27 | 0.34 | 1.04 | 0.42-2.61 | 0.93 | 0.81 | 0.48-1.39 | 0.45 | 0.45 | 0.65 |
| Platelet | 1.04 | 0.81-  1.32 | 0.76 | 1.25 | 0.79-1.98 | 0.34 | 1.00 | 0.75-1.35 | 0.97 | 0.78 | 0.43 |

Table A2: Presents the results of the conditional logistic regression analysis, displaying odds ratios (OR), confidence intervals (CI), p-values, and z-values.

Abbreviations: AMI = acute myocardial infarction; CKD = chronic kidney disease; AFib = atrial fibrillation; AFL = atrial flutter; AARAS = agents acting on the renin–angiotensin system; CVD exposure = includes atherosclerosis, AMI, stroke, hypertension, CKD, AFib/AFL, heart failure, and hypercholesterolemia.

Notes: * = p-value < 0.05; NA = too little variation or few observations. ´Composite CVD exposure´ consists of = AFib/AFL, AMI, atherosclerosis, CKD, heart failure, hypercholesterolemia, hypertension and stroke. ´Clinical CVD´ consists of = AFib/AFL, AMI, heart failure, atherosclerosis and stroke. ´Subclinical CVD´ consists of = CKD, hypercholesterolemia and hypertension.
